# Diagnostic accuracy of sarcopenia screening tools in low-income older adults in Amazonas, Brazil

**DOI:** 10.1007/s40520-025-03180-8

**Published:** 2025-10-30

**Authors:** Alex Barreto de Lima, Reshma Aziz Merchant, Duarte Henrinques-Neto

**Affiliations:** 1 Physical Education Course, Nilton Lins University, Amazonas, Brazil; 2 Skeletal Muscle Assessment Laboratory (LABSIM), Department of Physical Education, School of Technology and Sciences, São Paulo State University (UNESP), Presidente Prudente, Brazil; 3 Division of Geriatric Medicine, Department of Medicine, National University Hospital, Singapore, Singapore; 4 Department of Medicine, Yong Loo Lin School of Medicine, National University of Singapore, Singapore, Singapore; 5 Research Center in Sports Sciences, Health Sciences, and Human Development (CIDESD), University of Maia (UMaia), Av. Carlos Oliveira Campos - Castelo da Maia, Maia, 4475-690 Portugal

**Keywords:** Sarcopenia screening ishii test SarSA-Mod SARC-F SARC-Calf SARC-F + AC SARC-Calf + AC

## Abstract

**Background:**

Sarcopenia is recognized as an important geriatric condition that is both a consequence of and a contributor to various non-communicable diseases. It is a public health priority to improve early detection. However, there is still no consensus on the optimal diagnostic tools.

**Aims:**

This study evaluated the diagnostic performance of six sarcopenia screening tools - SARC-F, SARC-Calf, SARC-F + AC, SARC-Calf + AC, Ishii test, and SarSA-Mod in low-income older adults in Brazil.

**Methods:**

Sarcopenia was diagnosed using the European Working Group on Sarcopenia in Older People (EWGSOP2) and Sarcopenia Definitions and Outcomes Consortium (SDOC) criteria. Muscle mass was estimated through the Lee equation. The specificity, sensitivity, and receiver operating characteristic (ROC) curve analyses were calculated for the screening tools.

**Results:**

The study included 312 participants (200 women, mean age 72.4 ± 8.1 years). The prevalence of sarcopenia in men was 30.4% (EWGSOP2) and 75.0% (SDOC). In women, the prevalence was 28.5% (EWGSOP2) and 58.0% (SDOC). For men, the sensitivity/specificity of the Ishii test, SarSA-Mod, SARC-F, SARC-Calf, SARC- F + AC, and SARC-Calf + AC in screening for sarcopenia based on EWGSOP2 criteria were 69.2%/97.1%, 39.7%/94.1%, 50.0%/72.5%, 76.9%/38.2%, 85.9%/41.2% and 59.0%/67.7% respectively. Among women, these values were 52.5%/100%, 37.8%/70.2%, 83.2%/68.4%, 66.4%/69.5%, 87.4%/36.8% and 58.7%/63.2% respectively. the AUCs of SARC-F, SARC-Calf, SARC-F + AC, SARC-Calf + AC, SarSA-Mod and Ishii test in men, were 0.558, 0.576, 0.635, 0.633, 0.669, and 0.831, respectively. In women, the AUCs of SARC-F, SARC-Calf, SARC-F + AC, SARC-Calf + AC, SarSA-Mod and Ishii test were 0.574, 0.613, 0.621, 0.609, 0.540, and 0.762, respectively.

**Conclusions:**

The Ishii test showed the highest diagnostic accuracy for EWGSOP2-defined sarcopenia in community-dwelling older Brazilians.

**Supplementary Information:**

The online version contains supplementary material available at 10.1007/s40520-025-03180-8.

## Introduction

Muscle health is paramount due to its multifaceted roles in the human body [1]. It is defined as the state of optimal muscle mass, strength, and function [2]. Healthy muscles are fundamental for locomotion, postural support, and overall metabolic regulation [3]. Beyond these physical functions, muscles act as endocrine organs, releasing myokines, cytokines and other peptides during contractions. These molecules, such as irisin, interleukin-6, and brain-derived neurotrophic factor (BDNF), play crucial roles in healthy ageing and reducing long term morbidity and mortality [4–6].

Sarcopenia, defined as the progressive loss of muscle mass, strength, and physical function due to aging, is associated with an increased risk of adverse clinical outcomes, including disability, falls, and mortality [7]. It is also closely associated with chronic conditions such as osteoporosis, diabetes, hypertension, dyslipidemia, and many others [8–10]. The prevalence of sarcopenia in community-dwelling older adults ranges between 10 and 27% being highest in Oceania and lowest in Europe [11, 12]. In Africa, prevalence averaged 25.7% (7–48%), peaking in Cameroon [13]. The European DO-HEALTH study, involving older adults from four countries, identified a prevalence of sarcopenia of 2.0% using the Sarcopenia Definitions and Outcomes Consortion (SDOC) criteria, compared to 0.7% using the European Working Group on Sarcopenia in Older People (EWGSOP) 2 criteria, indicating that the SDOC may result in higher prevalences, even in populations from developed countries [14].

In Brazil, EWGSOP2-based prevalence ranges from 4.3% in the Federal District [15] to 18% in Rio de Janeiro [16], with intermediate rates in other major cities [17–20]. A study of 321 community-dwelling older adults reported SDOC-based prevalence of 1.6–7.6% and a 2.5-fold higher risk among malnourished individuals [21]. Sarcopenia has emerged as a significant public health concern due to its association with metabolic conditions, substantial medical care demands and the high costs of rehabilitation it imposes on both families and society [10]. Early-stage sarcopenia is difficult to diagnose due to the absence of clear symptoms. Physical disabilities and impairments generally become apparent only during its advanced stages when it is no longer reversible [22].

Early detection and diagnosis of sarcopenia are vital for effective prevention and management, especially in older populations. Timely identification allows for the implementation of individualized and targeted public health interventions, such as nutritional optimization and regular physical activity, to reduce adverse outcomes and support healthy aging [23]. Although sarcopenia is acknowledged as a diagnosis under the International Classification of Diseases (ICD) [24], its operational definition remains a topic of ongoing debate and discussion [25]. Currently, the consensus definition of sarcopenia from The European Working Group on Sarcopenia in Older People published in 2019 (EWGSOP2) is the most widely used in clinical practice [26]. The EWGSOP2 defines sarcopenia based on the presence of both low muscle strength and low muscle mass [26]. However, debates persist regarding the feasibility, reproducibility, and validity of these criteria for defining sarcopenia. Recently, the Sarcopenia Definition and Outcomes Consortium (SDOC) [27] advocated for an alternative approach, suggesting that sarcopenia should be defined by the presence of low physical performance alongside low muscle strength, shifting focus away from low muscle mass.

Researchers have developed various tools for early screening of sarcopenia, which generally fall into two categories. The first category comprises questionnaire-based screening tools, including the Strength, Assistance with Walking, Rising from a Chair, Climbing Stairs, and Falls questionnaire (SARC-F) [28], SARC-F combined with Calf Circumference measurement (SARC-Calf) [29], SARC-F plus Appendicular Circumference adjustment (SARC-F + AC), and SARC-Calf plus Appendicular Circumference adjustment (SARC-Calf + AC) [30], which are primarily based on the participants’ self-reporting and objective measures. The second category includes equation models, such as the Ishii test [31] and the sarcopenia scoring assessment model (SarSA-Mod) [32], which utilize calculations derived from physical examination findings to generate a diagnostic score.

The modified screening tools mentioned have not been simultaneously compared across different countries. The Ishii test, the first equation model specifically designed to detect sarcopenia, has been extensively validated across diverse populations [33, 34], but requires grip strength (HGS) measurements. In contrast, the SarSA-Mod, developed in 2021, provides high accuracy based on age, weight, and calf circumference (CC) and is more convenient than the Ishii test because it does not require HGS measurements [32]. To the best of our knowledge, the diagnostic effectiveness of SarSA-Mod has not yet been evaluated in Brazil.

Based on the considerations above, the primary objective of the study was to comprehensively compare the accuracy of different screening instruments to identify the most appropriate and effective tools for detecting sarcopenia among low-income, community-dwelling older adults in Brazil. The EWGSOP2 and SDOC criteria were used as diagnostic reference standards for sarcopenia.

## Methods

### Study design

This study utilized a cross-sectional design with a convenience sample of 312 community-dwelling older adults (64% women) residing in the city of Novo Aripuanã, located in Amazonas, Northern Brazil. Older adults living in rural areas were excluded from the study due to difficulty accessing the evaluation site (distance and necessary means of transport). Participants were included based on the following criteria: (a) age 60 years or older and living in the urban area of the city; (b) independence in performing activities of daily living; (c) no contraindications for physical exertion (stroke, neurological diseases, unstable chronic conditions); and (d) absence of chest pain, angina pectoris, or significant joint pain that could limit mobility [34]. Prior to data collection, all procedures and potential risks were thoroughly explained to participants, and informed consent forms were obtained from all individuals involved in the study. This project was approved by the Ethics and Research Committee of the Amazonas State University, following the Declaration of Helsinki [35] and Resolution 466/12 of the National Health Council [36] under ethics number: CAAE 74055517.9.0000.5016/Referee 2.281.400.

### Socioeconomic status

The sociodemographic variables were assessed using the questionnaire from the Brazilian Association of Research Companies [37]. This questionnaire classifies individuals into five social classes, spanning from class A (representing highest purchasing power) to class E (indicative of lowest purchasing power), determined by factors such as ownership of consumer goods, the educational level of the household head, and access to public services.

### Anthropometric measurements and body composition

Anthropometric measures included body mass, body height, mid-upper arm circumference (MUAC) and CC. Body mass index (BMI) was calculated by dividing body mass by body height squared (kg/m²). All measurements adhered to the standards outlined by the International Society for the Advancement of Kinanthropometry – ISAK [38] and were conducted by qualified physical education teachers with expertise in obtaining accurate.

### Sarcopenia definitions

According to the definition of EWGSOP2, the diagnosis of sarcopenia was made through a combined assessment of muscle strength, muscle mass and physical performance [26].

### Evaluation of muscle mass

The use of indirect methods to assess muscle mass (MM) was necessary due to the study population’s remote location, where access to advanced healthcare resources such as dual-energy X-ray absorptiometry (DXA) or magnetic resonance imaging (MRI) is limited or unavailable.

The MM was estimated using the through Lee equation: MM = Height(m) x (0.00744 x skinfold upperarm^2^ + 0.00088 x skinfold thigh^2^ + 0.00441 x calf girths^2^) + 2.4*sex − 0.048*age + race + 7.8, where R^2^ = 0.91, P: < 0.0001, and SEE = 2.2 kg; sex = 0 for female and 1 for male, race = 0 for white and Hispanic [39]. Skeletal muscle mass index (SMMI) was calculated dividing skeletal muscle mass by height squared [26].

### Muscle strength assessment

The HGS was measured using a dynamometer (Sensun Weighing Apparatus Group Ltd., Guangdong, China, USA) [40]. According to the EWGSOP2 criteria (11), HGS values below 27 kg for men and 16 kg for women were used to define low muscle strength [26]. The SDOC criteria defined low muscle strength as HGS below 35.5 kg for men and 20 kg for women [27]. Participants were seated in a chair with one arm resting over the corner of a small table, shoulder relaxed, elbow at a 90° angle, and hand in a neutral position without support from the Table [41]. Throughout the measurement, the arm being assessed was not supported on any surface [42]. The HGS was measured twice alternately for each hand, and the highest value recorded across the four measurements was used. Results were expressed in kilograms (kg).

### Physical performance

The standard 4-m speed test (4-MGS) was employed to evaluate physical performance. Participants were instructed to walk the designated distance at their usual walking speed, and the time taken to complete the walk was recorded and expressed in meters per second. A cutoff value of ≤ 0.8 m/s was used to indicate poor gait performance [26, 42]. If necessary, canes or walkers were permitted during this test. However, in this study, no participant needed to use a cane or walker to perform the test, as everyone walked normally, without any physical limitations. The 4-MGS is just one of several resources available to assess gait slowness and sarcopenia severity [43, 44].

Five times sit-to-stand test (5STS) was administered in accordance with standardized protocols [26, 45]. Participants were instructed to stand up as quickly as possible from a seated position starting with their full body weight supported on the chair until reaching a fully upright stance, with legs fully extended and arms crossed over the chest. The evaluator first demonstrated the procedure, and no lower limb physical tests were performed immediately prior to the 5STS to avoid fatigue-related interference with performance. The 5STS records the time, in seconds, required to complete five consecutive sit-to-stand movements. According to EWGSOP2 criteria, a time greater than 15 s was considered indicative of impaired performance [26].

### Sarcopenia screening tools

We used the SARC-F, SARC-CalF, SARC-F + AC, SARC-Calf + AC, Ishii test and SarSA-Mod for sarcopenia case-finding were evaluated (see Table 1).

**Table 1:** Sarcopenia case-finding tools with the recommended cut-off points for suspicion of sarcopenia

Screening tool	Components	Description
SARC-F	5 Items: Strength, Assistance in walking, Rise from a chair, Climb stairs and Falls	Subjective questionnaire: each item scored from 0 to 2 (total score from 0 to 10). Score ≥4 suggests risk of sarcopenia.
SARC-CalF	SARC-F + Calf Circumference (CC)	Combines the SARC-F with objective CC measurement. Adds 10 points to the SARC-F if CC < 34 cm (men) or < 33 cm (women). Score ≥11 indicates risk.
SARC-F + AC	SARC-F + Arm Circumference (AC)	SARC-F + AC score if AC is ≤29.5/≤28.4 cm in men/women, scores 10 points, otherwise 0 points are scored. SARC-F+AC total score is 0–20 points, and patients with a total score of ≥7 points can be judged to be at risk of sarcopenia
SARC-CalF + AC	SARC-F + Calf Circumference (CC) + Arm Circumference (AC)	The total of SARC-Calf+AC score is 30, and ≥12 points was classified as defining sarcopenia.
The Ishii Test	The score for males is calculated as follows: [0.62 × (ag e−64) −3.09× (grip strength−50) - 4.64×(CC−42)].The female score is calculated as follows: [0.8 × (age − 64) − 5. 09 × (grip strength − 34) − 3.28 × (CC − 42)].	A score of ≥105 points is the diagnostic cutoff for sarcopenia in males and ≥120 points in females
The SarSA-Mod	The score for males is calculated as follows: [1.4 × age) − 1. 2 × CC − (0.5 × weight) − 37.42].The female score is calculated as follows: [0.2 × age - 1.7 × CC − (weight + 92.56)]	The recommended cutoff points of −19.07 and −14.19 were selected for males and females, respectively.

The SARC-F questionnaire was developed to screen for sarcopenia in 2013 [28]. The study used the version translated into Brazilian Portuguese by Barbosa-Silva et al. [29]. It is a questionnaire that addresses five domains: strength, walking aids, difficulty getting up from a chair, difficulty climbing stairs and falls. Each domain has a question, and the answer is scored from 0 to 2 points for each item [28]. The total score ranges from 0 to 10, with ≥ 4 points indicating a high risk of sarcopenia.

The SARC-CalF questionnaire consists of the SARC-F added with a measurement of calf circumference [29]. The SARC-CalF score ranges from 0 to 20 points, with a score ≥ 11 points being suggestive of sarcopenia [29]. Men and women with calf circumferences less than 34 cm and 33 cm, respectively (suggesting low muscle mass), receive a 10-point increase over the original SARC-F score [29, 46].

The SARC-F + AC and SARC-Calf + AC were developed in 2021 by augmenting the SARC-F with arm circumference (AC) and CC. In these, a male AC of ≤ 29.5 cm or a female AC of ≤ 28.4 cm is assigned 10 points, otherwise, 0 points are given [30]. The scoring for the remaining items remains consistent. The SARC-F + AC has a total score ranging from 0 to 20 points, with scores of ≥ 7 points indicating a risk of sarcopenia. In contrast, the SARC-Calf + AC has a total score of 30 points, and individuals with scores ≥ 12 points are classified as having sarcopenia [30].

The Ishii test is an equation-based tool used to estimate the risk of sarcopenia, incorporating age, HGS, and CC as key factors [31]. For males, the score is calculated using the following formula: score = [0.62 x (age-64)-3.09 x (grip strength-50)-4.64 x (CC-42)]. For females, the score is calculated as follows: Score = [0.8 x (age − 64) − 5.09 x (grip strength − 34) − 3.28 x (CC-42)]. According to Ishii et al., a score of ≥ 105 points is used as the diagnostic cutoff for sarcopenia in males, while a score of ≥ 120 points serves as the threshold for females.

SarSA-Mod is a predictive equation developed to estimate the the risk of sarcopenia based on age, weight, and CC [32]. It was [0.2 x age − 1.7 x CC - (weight + 92.56)] for women and [1.4 x age) − 1.2 x CC - (0.5 x weight) − 37.42] for men. The recommended cutoff points for diagnosing sarcopenia are − 19.07 for men and − 14.19 for women, respectively.

### Statistical analysis

Statistical analyses were performed using SPSS v28.0 (SPSS Statistics; IBM, Armonk, NY) and MedCalc Statistical Software v19.20 (MedCalc Software bvba, Ostend, Belgium). Statistical analyses were stratified by gender. The t Student’s test and Chi-square test were used to compare continuous and categorical variables in the baseline characteristics of the study population, respectively. The sensitivity, specificity, positive predictive value (PPV), negative predictive value (NPV), and accuracy of the six sarcopenia screening tools were calculated. Receiver operating characteristic (ROC) curves were generated to assess and compare the diagnostic performance of the screening tools, with the area under the curve (AUC) used to quantify their accuracy. Commonly, an AUC ranging from 0 to 0.5 indicates no diagnostic value; 0.5–0.7, low diagnostic value; 0.7–0.9, intermediate diagnostic value; and 0.9–1, high diagnostic value [47]. Differences between ROC curves were analyzed using the DeLong method (50). The optimal cut-point was estimated by Youden Index [48]. A *p*-value of < 0.05 was considered statistically significant.

## Results

Participant characteristics, stratified by gender, are detailed in Table 2. The study population consisted of 112 men (35.9%) and 200 women (64.1%). Based on the EWGSOP2 criteria, 30.4% of men and 28.5% of women were diagnosed with sarcopenia. Statistically significant differences were observed in body mass, body height, muscle mass, skeletal muscle mass index, calf circumference, muscle strength, and Ishii test scores.

**Table 2:** Descriptive characteristics of participants as mean ± standard deviation

Characteristics	Male (n = 112)	Female (n = 200)	p-value	
Age, years	73.07±7.31	72.39±8.09	0.458	
Socioeconomic status class, n (%)			0.615	
C	5 (4.5)	13 (6.5)		
D/E	107 (95.5)	187 (93.5)		
Body Mass, kg	69.29±11.61	60.52±12.18	<0.001	
Body Height, cm	159.99±8.26	150.10±5.67	<0.001	
BMI, kg/m^2^	27.08±4.64	26.76±4.65	0.566	
Muscle mass, kg	23.65±3.55	17.73±3.61	<0.001	
SMMI, kg/m^2^	9.23±1.16	7.84±1.39	<0.001	
MUAC, cm	3.48±4.79	2.65±4.42	0.066	
Calf circumference, cm	33.8±3.0	32.2±3.5	<0.001	
Handgrip strength, kg	31.41±8.86	19.33±5.87	<0.001	
5x chair-stand test, s	11.38±3.87	12.83 ± 4.49	0.002	
Gait speed, m/s	1.19 ±0.35	1.03 ±0.35	<0.001	
Sarcopenia Diagnosis				
Ishii test, score	100.40±36.56	113.02±38.49	0.002	
SarSA-Mod, score	-10.40±15.02	-8.31±17.88	0.136	
Sarcopenia				
EWGSOP2, n (%)	34 (30.4)	57 (28.5)	0.729	
SDOC, n (%)	84 (75.0)	116 (58.0)	0.003	
SARC-F, n (%)			0.061	
<4	98 (87.5)	158 (79.0)		
>4	14 (12.5)	42 (21.0)		
SARC-CalF, n (%)			0.029	
<11	81 (72.3)	120 (60.0)		
>11	31 (27.7)	80 (40)		
SARC-F+AC, n (%)			0.554	
<7	87 (77.7)	161 (80.5)		
>7	25 (22.3)	39 (19.5)		
SARC-CalF+AC, n (%)			0.785	
<12	57 (50.9)	105 (52.5)		
>12	55 (49.1)	95 (47.5)		

Note: BMI, body mass index; SMMI, skeletal muscle mass index; MUAC, mid-upper arm circumference; EWGSOP2 European Working Group on Sarcopenia in Older People; SDOC, Sarcopenia Definition and Outcomes Consortium; SARC-F, A Simple Questionnaire to Rapidly Diagnose Sarcopenia

The results of sensitivity/specificity analyzes and AUCs of the Ishii test, SarSA-Mod, SARC-F, SARC-Calf, SARC-F + AC and SARC-Calf + AC based on EWGSOP2 2019 criteria are shown in Table 3.

**Table 3:** Comparison between the screening methods to according in EWGSOP2

Men	Area under curve	Sensitivity, (%)	Specificity, (%)	PPV, (%)	NPV, (%)
SARC-F	0.558 (0.438–0.678)^f^	50.0 (23.0–77.0)	72.5 (62.5–81.0)	20.6 (12.3–32.4)	91.0 (85.6–94.6)
SARC-CalF	0.576 (0.458–0.694)^f^	76.9 (66.0–85.7)	38.2 (22.2–56.4)	74.1 (68.1–79.3)	41.9 (28.6–56.6)
SARC-F+AC	0.635 (0.517–0.753)^f^	85.9 (76.8–92.7)	41.2 (24.7–59.3)	77.0 (71.4–81.8)	56.0 (39.2–71.5)
SARC-CalF+AC	0.633 (0.522–0.745)^f^	59.0 (47.3–70.0)	67.7 (49.5–82.6)	80.7 (71.3–87.6)	41.8 (33.6–50.6)
SarSA-Mod	0.669 (0.569–0.770)^e,f^	39.7 (28.8–51.5)	94.1 (80.3–99.3)	93.9 (79.7–98.4)	40.5 (35.8–45.4)
Ishii test	0.831 (0.756–0.907)^a–e^	69.2 (57.8–79.2)	97.1 (84.7–99.9)	98.2 (88.6–99.7)	57.9 (49.5–65.9)
Women					
SARC-F	0.574 (0.483–0.665)^f^	83.2 (76.1–89.0)	68.4 (54.8–80.1)	86.9 (81.8–90.7)	61.9 (52.0–70.9)
SARC-CalF	0.613 (0.525–0.700)^f^	66.4 (58.1–74.1)	69.5 (58.4–79.2)	79.2 (72.9–84.3)	54.3 (47.5–60.9)
SARC-F+AC	0.621 (0.530–0.712)^f^	87.4 (80.8–92.4)	36.8 (24.5–50.7)	77.6 (73.8–81.0)	53.9 (40.2–66.9)
SARC-CalF+AC	0.609 (0.523–0.696)^f^	58.7 (50.2–66.9)	63.2 (49.3–75.6)	80.0 (73.5–85.2)	37.9 (31.6–44.6)
SarSA-Mod	0.540 (0.452–0.627)^f^	37.8 (29.8–46.3)	70.2 (56.6–81.6)	76.1 (66.9–83.3)	31.0 (26.7–35.7)
Ishii test	0.762 (0.699–0.826)^a–e^	52.5 (43.9–60.9)	100 (93.7–100.0)	100	45.6 (41.4–49.9)

^a^Significantly different relative to SARC-F^b^Significantly different relative to the SARC-CalF^c^Significantly different relative to the SARC-F+AC^d^Significantly different relative to the SARC-CalF+AC^e^Significantly different relative to the SarSA-Mod^f^Significantly different relative to the Ishii test

The comparison of the results in Table 3 across the different screening instruments showed that, among men, the Ishii test exhibited the highest discriminative power, with an area under the curve (AUC) of 0.831 (95% CI: 0.756–0.907), a specificity of 97.1%, and a sensitivity of 69.2%. In contrast, the SarSA-Mod demonstrated the lowest discriminative ability, with an AUC of 0.669 (95% CI: 0.569–0.770), a specificity of 94.1%, and a sensitivity of 39.7%. Among the SARC-F–based methods, the version combined with arm circumference (SARC-F + AC) showed the best performance (AUC = 0.635), with high sensitivity (85.9%) and moderate specificity (41.2%), while SARC-F, SARC-CalF, and SARC-CalF + AC presented lower AUC values ranging from 0.558 to 0.633Among women, the Ishii test also demonstrated the best overall performance, with an AUC of 0.762 (95% CI: 0.699–0.826), showing perfect specificity (100%) but moderate sensitivity (52.5%). The SARC-F, SARC-F + AC, SARC-CalF, and SARC-CalF + AC instruments exhibited moderate discriminative ability, with AUC values ranging between 0.57 and 0.62. Similar to the findings in men, the SarSA-Mod showed the lowest discriminative power among the tools, with an AUC of 0.540.

More details on NPV, PPV, and AUC are presented in Table 3.

The results of sensitivity/specificity analyzes and AUCs of the Ishii test, SarSA-Mod, SARC-F, SARC-Calf, SARC-F + AC and SARC-Calf + AC according to the SDOC criteria are shown in Table 4.

**Table 4:** Comparison between the screening methods to according in SDOC

Men	Area under curve	Sensitivity, (%)	Specificity, (%)	PPV, (%)	NPV, (%)
SARC-F	0.536 (0.416–0.656)^f^	92.9 (76.6–99.2)	14.3 (7.6–23.6)	26.5 (24.0–29.2)	85.7 (58.8–96.2)
SARC-CalF	0.613 (0.501–0.726)^f^	89.3 (71.8–97.7)	32.5 (22.7–43.7)	30.9 (26.8–35.2)	90.0 (74.7–96.5)
SARC-F+AC	0.625 (0.516–0.734)^f^	96.4 (81.7–99.9)	28.6 (19.2–39.5)	31.0 (27.9–34.4)	96.0 (77.3–99.4)
SARC-CalF+AC	0.661 (0.547–0.774)^f^	75.0 (55.1–89.3)	57.1 (45.9–67.9)	36.8 (29.6–44.7)	87.3 (77.9–93.0)
SarSA-Mod	0.661 (0.538–0.783)^f^	53.6 (33.9–72.5)	78.6 (68.3–86.8)	45.5 (32.8–58.7)	83.5 (77.0–88.5)
Ishii test	0.815 (0.736–0.895)^a–e^	96.4 (81.7–99.9)	66.7 (55.5–76.6)	49.1 (41.4–56.8)	98.3 (89.0–99.7)
Women					
SARC-F	0.527 (0.446–0.608)^f^	82.1 (72.3–89.7)	23.3 (15.9–32.0)	43.7 (40.2–47.2)	64.3 (50.6–76.0)
SARC-CalF	0.599 (0.519–0.678)^f^	71.4 (60.5–80.8)	48.3 (38.9–57.7)	50.0 (44.5–55.5)	70.0 (61.3–77.5)
SARC-F+AC	0.576 (0.497–0.655)^f^	89.3 (80.6–95.0)	25.9 (18.2–34.8)	46.6 (43.4–49.8)	76.9 (62.6–86.9)
SARC-CalF+AC	0.581 (0.501–0.661)^f^	61.9 (50.7–72.3)	54.3 (44.8–63.6)	49.5 (43.1–56.0)	66.3 (58.9–73.1)
SarSA-Mod	0.584 (0.503–0.665)^f^	45.2 (34.3–56.5)	71.6 (62.4–79.5)	53.5 (44.2–62.6)	64.3 (59.0–69.3)
Ishii test	0.885 (0.831–0.939)^a–e^	82.1 (72.3–89.7)	94.8 (89.1–98.1)	92.0 (83.9–96.2)	88.0 (82.2–92.1)

^a^Significantly different relative to SARC-F^b^Significantly different relative to the SARC-CalF^c^Significantly different relative to the SARC-F+AC^d^Significantly different relative to the SARC-CalF+AC^e^Significantly different relative to the SarSA-Mod^f^Significantly different relative to the Ishii test

The Ishii test consistently demonstrated the highest discriminative power for sarcopenia across both EWGSOP2 and SDOC criteria in men and women. Under EWGSOP2, it achieved the highest AUC (0.831 in men; 0.762 in women), with excellent specificity (97.1–100%) but moderate sensitivity (52.5–69.2%). In contrast, the SarSA-Mod showed the lowest performance (AUC 0.669 in men; 0.540 in women). Among SARC-F–based tools, SARC-F + AC performed best, particularly in men, with high sensitivity (85.9%) but low specificity (41.2%), while other variants had lower AUC values (0.558–0.633).

Similarly, under SDOC criteria, the Ishii test outperformed other tools, with AUCs of 0.815 in men and 0.885 in women, combining high sensitivity (82.1–96.4%) and good-to-excellent specificity (66.7–94.8%). SARC-F–based instruments, while highly sensitive (up to 96.4%), had poor specificity (14.3–28.6%), limiting their discriminative ability (AUC 0.536–0.625). Other tools, including SarSA-Mod and SARC-CalF + AC, showed moderate accuracy (AUC ~ 0.66), whereas SARC-CalF had the highest performance among non-Ishii tools in women (AUC 0.599).

## Discussion

This study systematically evaluated the diagnostic accuracy of six sarcopenia screening tools (SARC-F, SARC-Calf, SARC-F + AC, SARC-Calf + AC, Ishii test and SarSA-Mod) against the EWGSOP2 and SDOC criteria in low-income older Brazilians living in communities in the Amazonas. With aging population globally, timely detection of sarcopenia is crucial in reducing disability and preserving quality of life [10, 49]. However, screening remains challenging, and tools validated in high-income countries may over, or under-estimate sarcopenia prevalence when applied to low- and middle-income countries [50]. Few studies have directly compared existing traditional screening tools with the newer ones, and no universal consensus regarding the optimal tool.

The results demonstrated significant variability in diagnostic performance across tools, reinforcing the need to select instruments according to the target population and clinical setting. Consistent with prior research, the Ishii test showed the highest overall accuracy, with high specificity and moderate sensitivity under both EWGSOP2 and SDOC criteria, and particularly strong performance among women. Its superior discriminative power reflected in higher AUC values especially under SDOC, which directly captures musculoskeletal decline. These findings align with previous research validating the Ishii test across various older populations, further supporting its effectiveness and applicability in clinical practice [33, 34]. SarSA-Mod exhibited the lowest accuracy in our study, differing from findings in older Iranians [32], highlighting the importance of context-specific validation.

The SARC-F, originally developed as a simple screening tool for the detection of sarcopenia [28], is known for its high specificity (68.9–88.9%) but has lower sensitivity (28.9–55.3%) [46, 51–53]. Several modified versions of the SARC-F have been developed to address its limitations, with researchers working to enhance its sensitivity to varying degrees. For example, Barbosa-Silva et al. [29] incorporated CC into SARC-F, creating SARC-CalF, which significantly increased its sensitivity from 33.3 to 66.7% in Brazilian older adults. In contrast, the modified versions of SARC-F, including SARC-F + AC and SARC-Calf + AC, demonstrated improved sensitivity, a desirable feature in screening scenarios. However, this enhancement came at the expense of a significant reduction in specificity. For example, the sensitivity of SARC-F + AC in women was 87.4% (EWGSOP2) and 89.3% (SDOC), but its specificity fell to 36.8% and 25.9%, respectively. This indicates that these modified versions may overestimate the risk of sarcopenia, resulting in a higher number of false positives. This observation is consistent with findings by Zhou et al. [30] and Krzymińska-Siemaszko et al. [11], who also highlighted the challenge of balancing sensitivity and specificity in SARC-F modifications. Another noteworthy aspect is the variation in tool performance between genders. For instance, the sensitivity of the SARC-Calf was higher in men (76.9%) compared to women (66.4%), while specificity remained similar across genders. This disparity may be attributed to anatomical and functional differences between men and women, emphasizing the importance of incorporating gender-stratified approaches in sarcopenia screening [14, 27].

Despite its practicality, the SARC-F demonstrated limited effectiveness in detecting sarcopenia, consistent with previous studies highlighting its low sensitivity (generally below 30%) [28, 30]. In this study, the SARC-F showed moderate sensitivity in men and higher sensitivity in women, while specificity values were moderate for both genders. Although the SARC-F exhibited good negative predictive value in certain contexts, its use as a standalone tool may lead to underdiagnosis, particularly in populations with reduced functional awareness, such as older adults with low educational attainment or restricted access to healthcare services [46].

On the other hand, SarSA-Mod, which is based on age, weight and calf circumference, showed lower performance than expected, with sensitivity of only 39.7% (men) and 37.8% (women) according to EWGSOP2, and similar values in SDOC. While its specificity was relatively strong, especially in men, the tool’s limited sensitivity reduces its efficacy as a comprehensive screening method. Although SarSA-Mod shows promise due to its ease of use, as it does not rely on dynamometers, further validation and adaptations are necessary for Brazilian populations, particularly those in regions with unique sociocultural and anthropometric traits, like the Amazon [31].

The prevalence of sarcopenia observed in this study (30.4% EWGSOP2 and up to 75% SDOC among men) was higher than the world average (11–20%) [54], reflecting the vulnerability of the population studied. Factors such as socioeconomic category, health literacy, dietary and health access factors may have contributed to lower baseline muscle strength and functional capacity, indicating the need for public policies to prevent and detect sarcopenia early in disadvantaged communities.

Therefore, our findings reinforce the importance of using screening tools adjusted to the population context. In low-income populations, the use of tools that combine simple anthropometric measurements with functional questionnaires, such as the SARC-CalF or the SarSA-Mod, may be more effective and feasible than instruments based on self-report alone.

### Strengths and limitations

This study has several limitations that should be acknowledged. Firstly, the representativeness of the sample may not be fully representative of the low-income population in the interior of the Amazonas region (age group, gender, socioeconomic level, residential area), since we excluded participants living in rural areas, indigenous people and people from other municipalities, due to the fact that travel between cities is only possible by river, taking up to two days. Secondly, muscle mass analysis was not performed using the gold standard method (DXA), potentially increasing the error rate in identifying sarcopenia. However, estimation using the Lee equation method has been validated by whole-body MRI scans with R^2^ = 0.89, *p* < 0.001 [39]. Additionally, factors such as diet, physical activity, alcohol consumption, medication use, and the presence of non-communicable diseases were not assessed, which may introduce bias into the results.

Despite these limitations, our study has notable strengths. To our knowledge, it is the first study conducted in a population with such specific social, demographic, and biological characteristics. Accessing these populations was challenging and required travel by boat. Despite difficult material and logistical conditions, the tools analyzed showed high applicability due to their simplicity, low cost, and high sensitivity. Studies like this underscore the need to develop monitoring and public health promotion tools tailored to low-income populations.

## Conclusion

This study demonstrated that the accuracy of screening tools for sarcopenia varies considerably depending on the method used, the diagnostic criteria used and the sex of the individuals. Among the instruments evaluated, the Ishii test emerged as the most effective, excelling in sensitivity, specificity, and AUC. Its strong performance positions it as a promising tool for screening sarcopenia in low-income older populations. Although the modifications to the SARC-F improved sensitivity, the low specificity values compromise its applicability as a single screening tool. The SarSA-Mod, although simple and accessible, showed limited performance, especially among women.

Thus, the findings highlight the importance of selecting validated screening methods for specific populations, considering their clinical, social and cultural characteristics. The use of the Ishii test may be a viable strategy for sarcopenia screening in low-resource settings. Its use could aid in the early detection of sarcopenia and promote the implementation of effective preventative measures within the Brazilian public health framework.

## Supplementary Information

Below is the link to the electronic supplementary material.

Supplementary Material 1

Supplementary Material 2

Supplementary Material 3

## Data Availability

No datasets were generated or analysed during the current study.
