# Supplementary material for: Diagnostic accuracy of sarcopenia screening tools in low-income older adults in Amazonas, Brazil
Source: Aging Clin Exp Res. 2025 Oct 30;37(1):307. doi: 10.1007/s40520-025-03180-8 (PMC12575561; doi:10.1007/s40520-025-03180-8)
Supplement: Supplementary file 1 — Supplementary Material 1 [file 40520_2025_3180_MOESM1_ESM.doc]

| Table 1. Descriptive characteristics of participants as mean ± standard deviation | | | | |
| --- | --- | --- | --- | --- |
| Characteristics | Male (n = 112) | Female (n = 200) | p-value | |
| Age, years | 73.07±7.31 | 72.39±8.09 | 0.458 | |
| Socioeconomic status class, n (%) |  |  | 0.615 | |
| C | 5 (4.5) | 13 (6.5) |  | |
| D/E | 107 (95.5) | 187 (93.5) |  | |
| Body Mass, kg | 69.29±11.61 | 60.52±12.18 | <0.001 | |
| Body Height, cm | 159.99±8.26 | 150.10±5.67 | <0.001 | |
| BMI, kg/m^2^ | 27.08±4.64 | 26.76±4.65 | 0.566 | |
| Muscle mass, kg | 23.65±3.55 | 17.73±3.61 | <0.001 | |
| SMMI, kg/m^2^ | 9.23±1.16 | 7.84±1.39 | <0.001 | |
| MUAC, cm | 3.48±4.79 | 2.65±4.42 | 0.066 | |
| Calf circumference, cm | 33.8±3.0 | 32.2±3.5 | <0.001 | |
| Handgrip strength, kg | 31.41±8.86 | 19.33±5.87 | <0.001 | |
| 5x chair-stand test, s | 11.38±3.87 | 12.83 ± 4.49 | 0.002 | |
| Gait speed, m/s | 1.19 ±0.35 | 1.03 ±0.35 | <0.001 | |
| *Sarcopenia Diagnosis* |  |  |  | |
| Ishii test, score | 100.40±36.56 | 113.02±38.49 | 0.002 | |
| SarSA-Mod, score | -10.40±15.02 | -8.31±17.88 | 0.136 | |
| Sarcopenia |  |  |  | |
| EWGSOP2, n (%) | 34 (30.4) | 57 (28.5) | 0.729 |  |
| SDOC, n (%) | 84 (75.0) | 116 (58.0) | 0.003 |  |
| SARC-F, n (%) |  |  | 0.061 | |
| <4 | 98 (87.5) | 158 (79.0) |  | |
| >4 | 14 (12.5) | 42 (21.0) |  | |
| SARC-CalF, n (%) |  |  | 0.029 | |
| <11 | 81 (72.3) | 120 (60.0) |  | |
| >11 | 31 (27.7) | 80 (40) |  | |
| SARC-F+AC, n (%) |  |  | 0.554 | |
| <7 | 87 (77.7) | 161 (80.5) |  | |
| >7 | 25 (22.3) | 39 (19.5) |  | |
| SARC-CalF+AC, n (%) |  |  | 0.785 | |
| <12 | 57 (50.9) | 105 (52.5) |  | |
| >12 | 55 (49.1) | 95 (47.5) |  | |
| Note: BMI, body mass index; SMMI, skeletal muscle mass index; MUAC, mid-upper arm circumference; EWGSOP2 European Working Group on Sarcopenia in Older People; SDOC, Sarcopenia Definition and Outcomes Consortium; SARC-F, A Simple Questionnaire to Rapidly Diagnose Sarcopenia; | | | | |
