# Supplementary material for: Diagnostic accuracy of sarcopenia screening tools in low-income older adults in Amazonas, Brazil
Source: Aging Clin Exp Res. 2025 Oct 30;37(1):307. doi: 10.1007/s40520-025-03180-8 (PMC12575561; doi:10.1007/s40520-025-03180-8)
Supplement: Supplementary file 2 — Supplementary Material 2 [file 40520_2025_3180_MOESM2_ESM.doc]

| **Table** **2** - Comparison between the screening methods to according in EWGSOP2 | | | | | |
| --- | --- | --- | --- | --- | --- |
| Men | Area under curve | Sensitivity, (%) | Specificity, (%) | PPV, (%) | NPV, (%) |
| SARC-F | 0.558 (0.438-0.678)^f^ | 50.0 (23.0-77.0) | 72.5 (62.5-81.0) | 20.6 (12.3-32.4) | 91.0 (85.6-94.6) |
| SARC-CalF | 0.576 (0.458-0.694)^f^ | 76.9 (66.0-85.7) | 38.2 (22.2-56.4) | 74.1 (68.1-79.3) | 41.9 (28.6-56.6) |
| SARC-F+AC | 0.635 (0.517-0.753)^f^ | 85.9 (76.8-92.7) | 41.2 (24.7-59.3) | 77.0 (71.4-81.8) | 56.0 (39.2-71-5) |
| SARC-CalF+AC | 0.633 (0.522-0.745)^f^ | 59.0 (47.3-70.0) | 67.7 (49.5-82.6) | 80.7 (71.3-87.6) | 41.8 (33.6-50.6) |
| SarSA-Mod | 0.669 (0.569-0.770)^e,f^ | 39.7 (28.8-51.5) | 94.1 (80.3-99.3) | 93.9 (79.7-98.4) | 40.5 (35.8-45.4) |
| Ishii test | 0.831 (0.756-0.907)^a-e^ | 69.2 (57.8-79.2) | 97.1 (84.7-99.9) | 98.2 (88.6-99.7) | 57.9 (49.5-65.9) |
| Women |  |  |  |  |  |
| SARC-F | 0.574 (0.483-0.665)^f^ | 83.2 (76.1-89.0) | 68.4 (54.8-80.1) | 86.9 (81.8-90.7) | 61.9 (52.0-70.9) |
| SARC-CalF | 0.613 (0.525-0.700)^f^ | 66.4 (58.1-74.1) | 69.5 (58.4-79.2) | 79.2 (72.9-84.3) | 54.3 (47.5-60.9) |
| SARC-F+AC | 0.621 (0.530-0.712)^f^ | 87.4 (80.8-92.4) | 36.8 (24.5-50.7) | 77.6 (73.8-81.0) | 53.9 (40.2-66.9) |
| SARC-CalF+AC | 0.609 (0.523-0.696)^f^ | 58.7 (50.2-66.9) | 63.2 (49.3-75.6) | 80.0 (73.5-85.2) | 37.9 (31.6-44.6) |
| SarSA-Mod | 0.540 (0.452-0.627)^f^ | 37.8 (29.8-46.3) | 70.2 (56.6-81.6) | 76.1 (66.9-83.3) | 31.0 (26.7-35.7) |
| Ishii test | 0.762 (0.699-0.826)^a-e^ | 52.5 (43.9-60.9) | 100 (93.7-100.0) | 100 | 45.6 (41.4-49.9) |
| ^a^Significantly different relative to SARC-F  ^b^Significantly different relative to the SARC-CalF  ^c^Significantly different relative to the SARC-F+AC  ^d^Significantly different relative to the SARC-CalF+AC  ^e^Significantly different relative to the SarSA-Mod  ^f^Significantly different relative to the Ishii test | | | | | |
