# Supplementary material for: Diagnostic accuracy of sarcopenia screening tools in low-income older adults in Amazonas, Brazil
Source: Aging Clin Exp Res. 2025 Oct 30;37(1):307. doi: 10.1007/s40520-025-03180-8 (PMC12575561; doi:10.1007/s40520-025-03180-8)
Supplement: Supplementary file 3 — Supplementary Material 3 [file 40520_2025_3180_MOESM3_ESM.docx]

| **Table** 3 - Comparison between the screening methods to according in SDOC | | | | | |
| --- | --- | --- | --- | --- | --- |
| Men | Area under curve | Sensitivity, (%) | Specificity, (%) | PPV, (%) | NPV, (%) |
| SARC-F | 0.536 (0.416-0.656)^f^ | 92.9 (76.6-99.2) | 14.3 (7.6-23.6) | 26.5 (24.0-29.2) | 85.7 (58.8-96.2) |
| SARC-CalF | 0.613 (0.501-0.726)^f^ | 89.3 (71.8-97.7) | 32.5 (22.7-43.7) | 30.9 (26.8-35.2) | 90.0 (74.7-96.5) |
| SARC-F+AC | 0.625 (0.516-0.734)^f^ | 96.4 (81.7-99.9) | 28.6 (19.2-39.5) | 31.0 (27.9-34.4) | 96.0 (77.3-99.4) |
| SARC-CalF+AC | 0.661 (0.547-0.774)^f^ | 75.0 (55.1-89.3) | 57.1 (45.9-67.9) | 36.8 (29.6-44.7) | 87.3 (77.9-93.0) |
| SarSA-Mod | 0.661 (0.538-0.783)^f^ | 53.6 (33.9-72.5) | 78.6 (68.3-86.8) | 45.5 (32.8-58.7) | 83.5 (77.0-88.5) |
| Ishii test | 0.815 (0.736-0.895)^a-e^ | 96.4 (81.7-99.9) | 66.7 (55.5-76.6) | 49.1 (41.4-56.8) | 98.3 (89.0-99.7) |
| Women |  |  |  |  |  |
| SARC-F | 0.527 (0.446-0.608)^f^ | 82.1 (72.3-89.7) | 23.3 (15.9-32-0) | 43.7 (40.2-47.2) | 64.3 (50.6-76.0) |
| SARC-CalF | 0.599 (0.519-0.678)^f^ | 71.4 (60.5-80.8) | 48.3 (38.9-57.7) | 50.0 (44.5-55.5) | 70.0 (61.3-77.5) |
| SARC-F+AC | 0.576 (0.497-0.655)^f^ | 89.3 (80.6-95.0) | 25.9 (18.2-34.8) | 46.6 (43.4-49.8) | 76.9 (62.6-86.9) |
| SARC-CalF+AC | 0.581 (0.501-0.661)^f^ | 61.9 (50.7-72.3) | 54.3 (44.8-63.6) | 49.5 (43.1-56.0) | 66.3 (58.9-73.1) |
| SarSA-Mod | 0.584 (0.503-0.665)^f^ | 45.2 (34.3-56.5) | 71.6 (62.4-79.5) | 53.5 (44.2-62.6) | 64.3 (59.0-69.3) |
| Ishii test | 0.885 (0.831-0.939)^a-e^ | 82.1 (72.3-89.7) | 94.8 (89.1-98.1) | 92.0 (83.9-96.2) | 88.0 (82.2-92.1) |
| ^a^Significantly different relative to SARC-F  ^b^Significantly different relative to the SARC-CalF  ^c^Significantly different relative to the SARC-F+AC  ^d^Significantly different relative to the SARC-CalF+AC  ^e^Significantly different relative to the SarSA-Mod  ^f^Significantly different relative to the Ishii test | | | | | |
